# Supplementary figures and images for: The ecomorphology of the shell of extant turtles and its applications for fossil turtles
Source: PeerJ. 2020 Dec 22;8:e10490. doi: 10.7717/peerj.10490 (PMC7761203; doi:10.7717/peerj.10490)

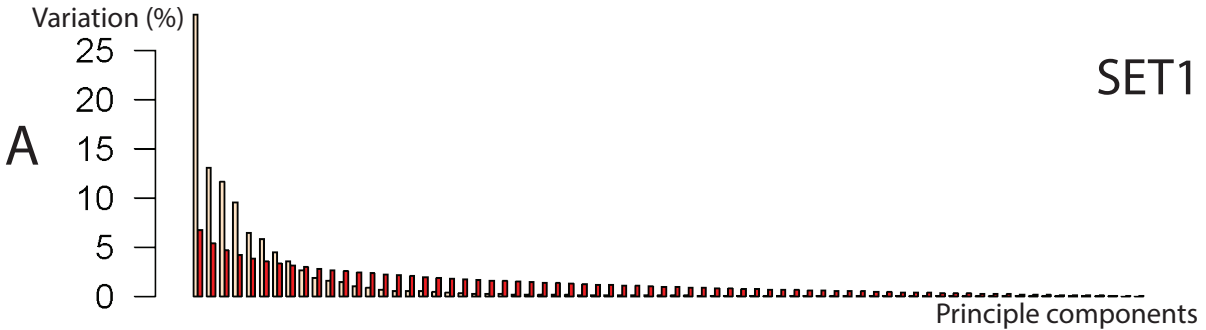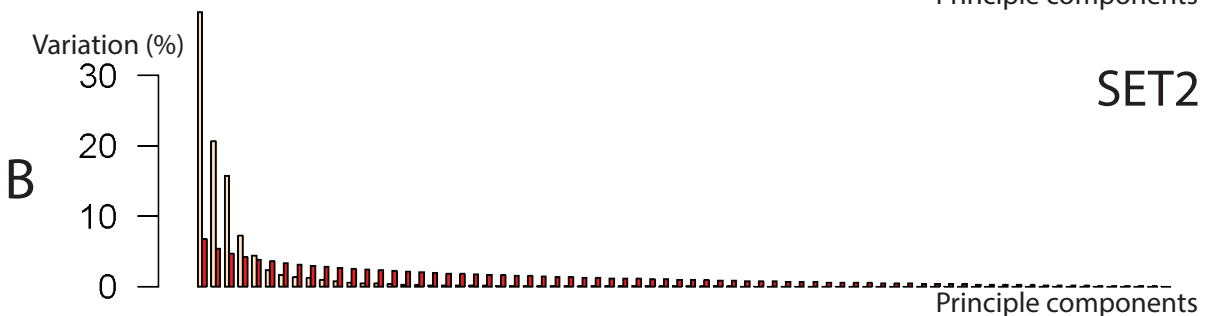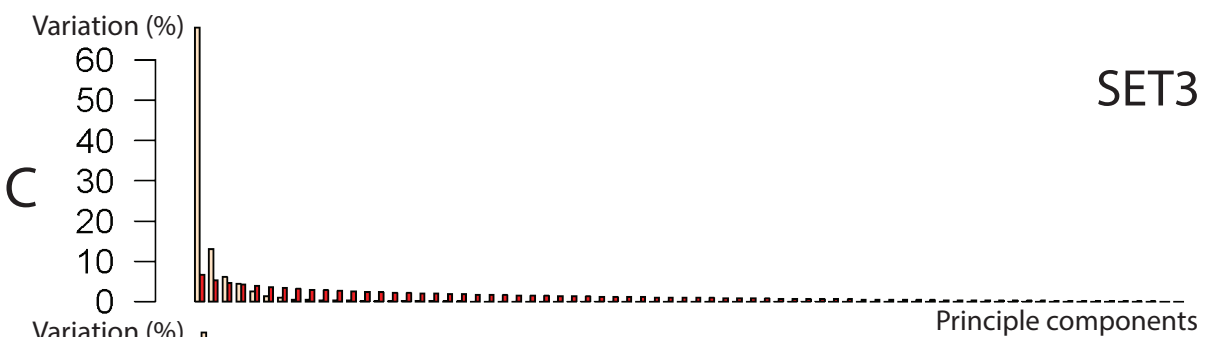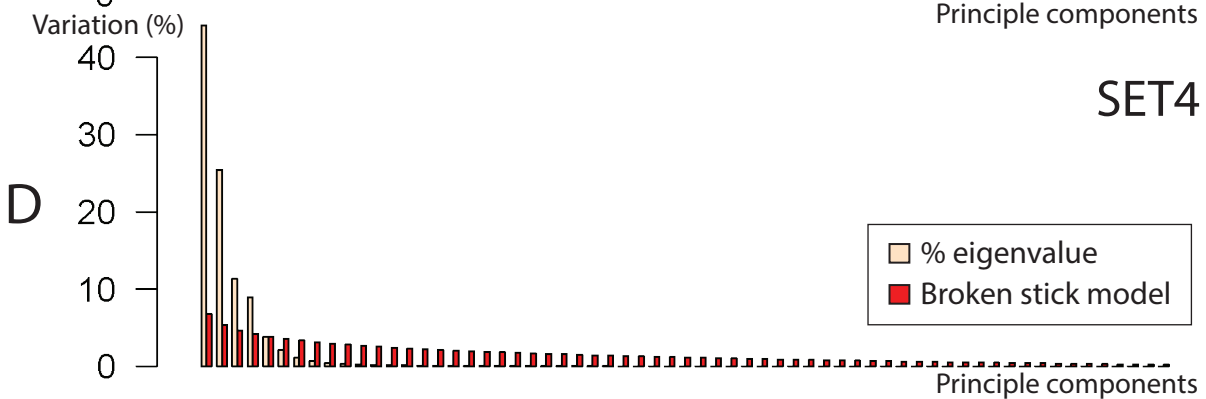

Supplement: Supplemental Information 1 — The y-axis represents the percentage of variation explained by each PC. (A) Model with all collected data, SET1. (B) Outline of the carapace, SET2. (C) Transverse cross section, SET3. (D) Longitudinal cross section, SET4. [file peerj-08-10490-s001.pdf]

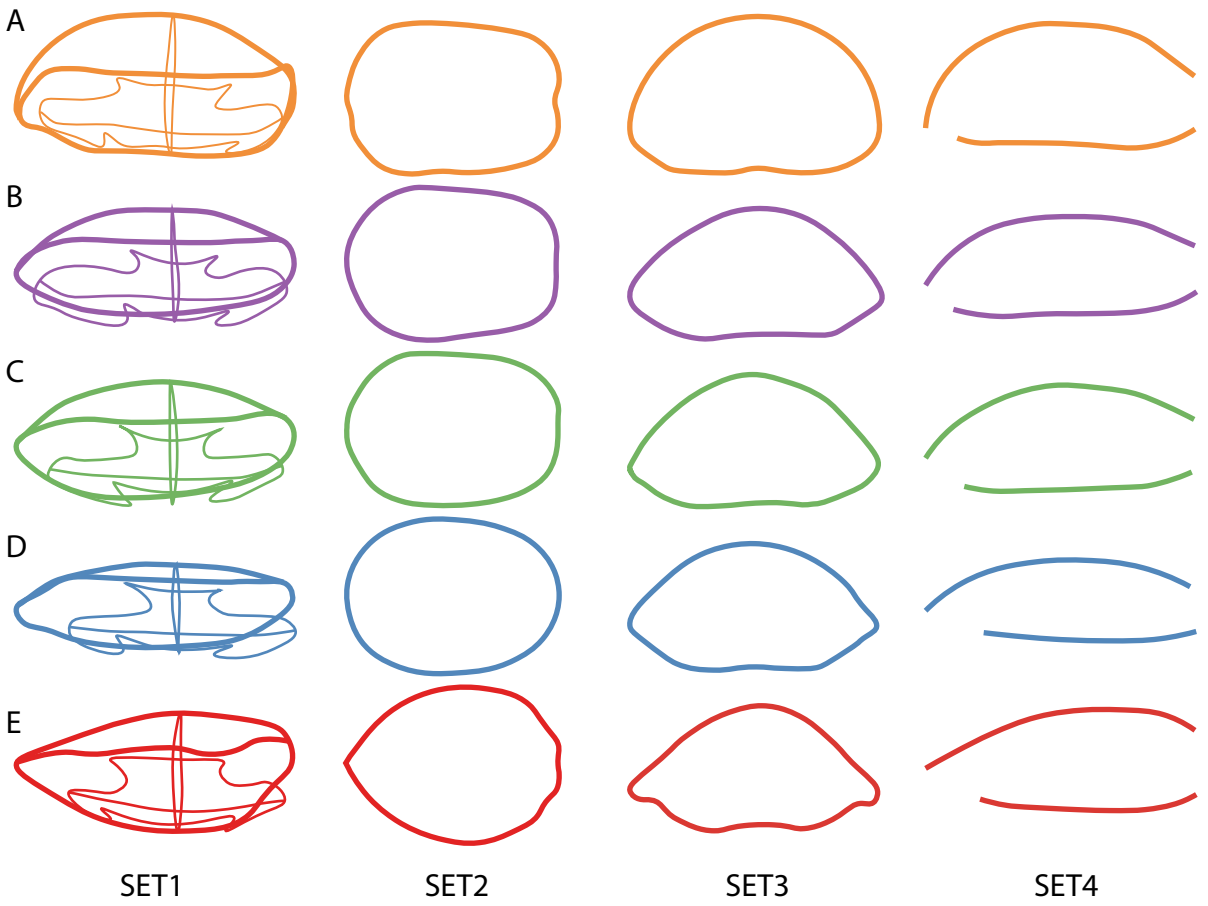

Supplement: Supplemental Information 2 — (A) “not webbed” (category 0). (B) “poorly webbed” (category 1). (C) “fully webbed” (category 2). (D) “extensive webbing” (category 3). (E) “flippers” (category 4). Mean shapes were computed using the function “mshape” from the R package geomorph. [file peerj-08-10490-s002.pdf]

**A**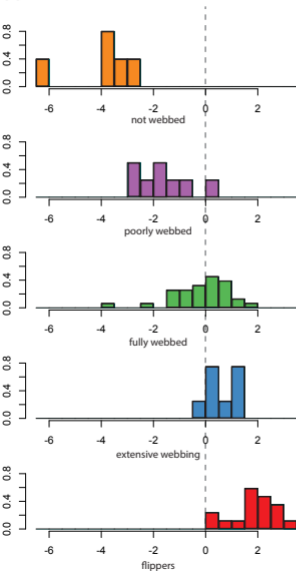**B**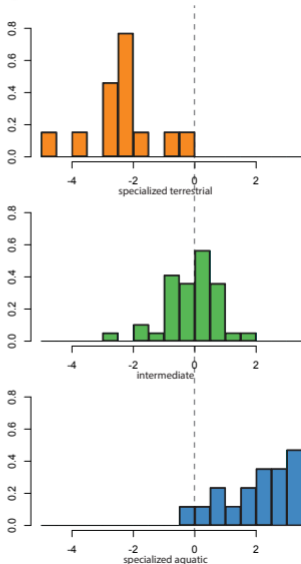

Supplement: Supplemental Information 3 — (A) The categories not-webbed and flippers are very distinct using 5 categories. The three intermediate categories are overlapping each other.(B) The categories terrestrial and aquatic are very distinct using 3 categories. The intermediate categories are overlapping the terrestrial range. [file peerj-08-10490-s003.pdf]

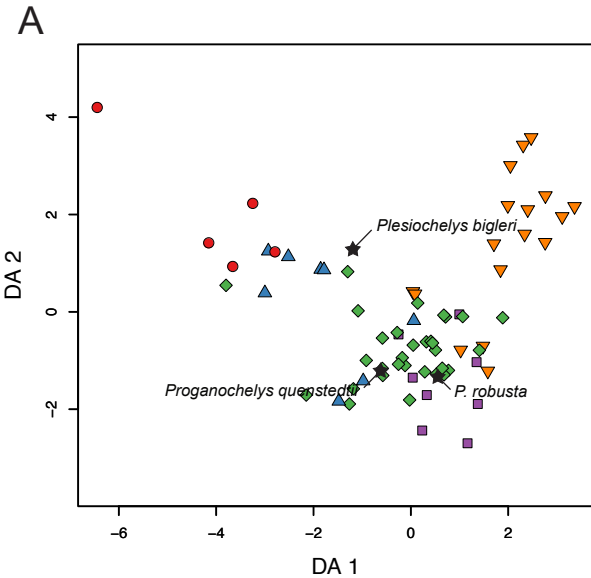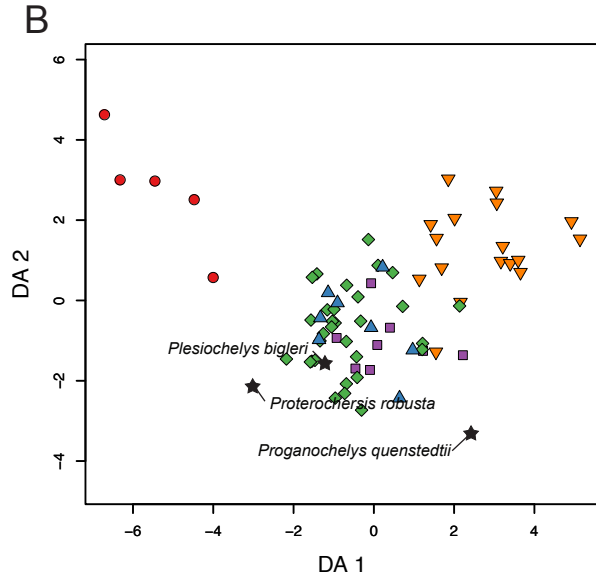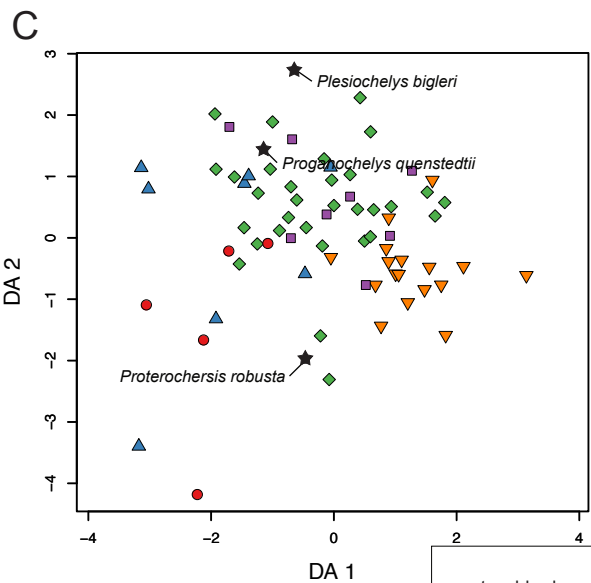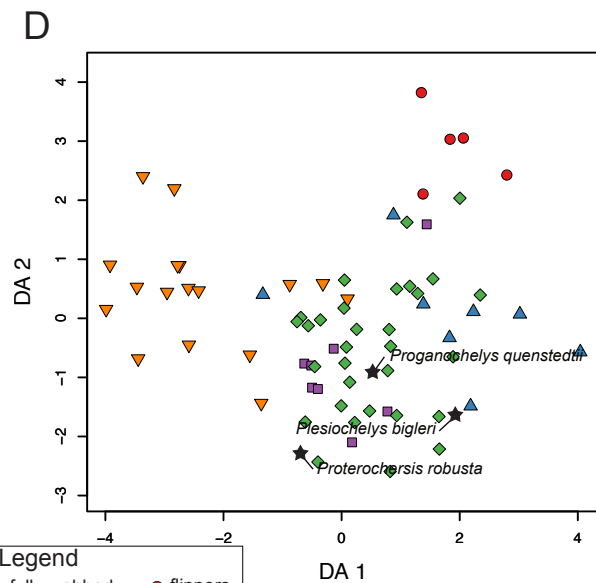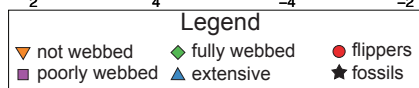

Supplement: Supplemental Information 4 — (A) All landmarks and curves, SET1. (B) Outline of the carapace, SET2. (C) Transverse cross-section, SET3. (D) Longitudinal cross-section, SET4. Phylogenetic tree used in the analysis is available in Supplementary Files, Fig. S5 [file peerj-08-10490-s004.pdf]

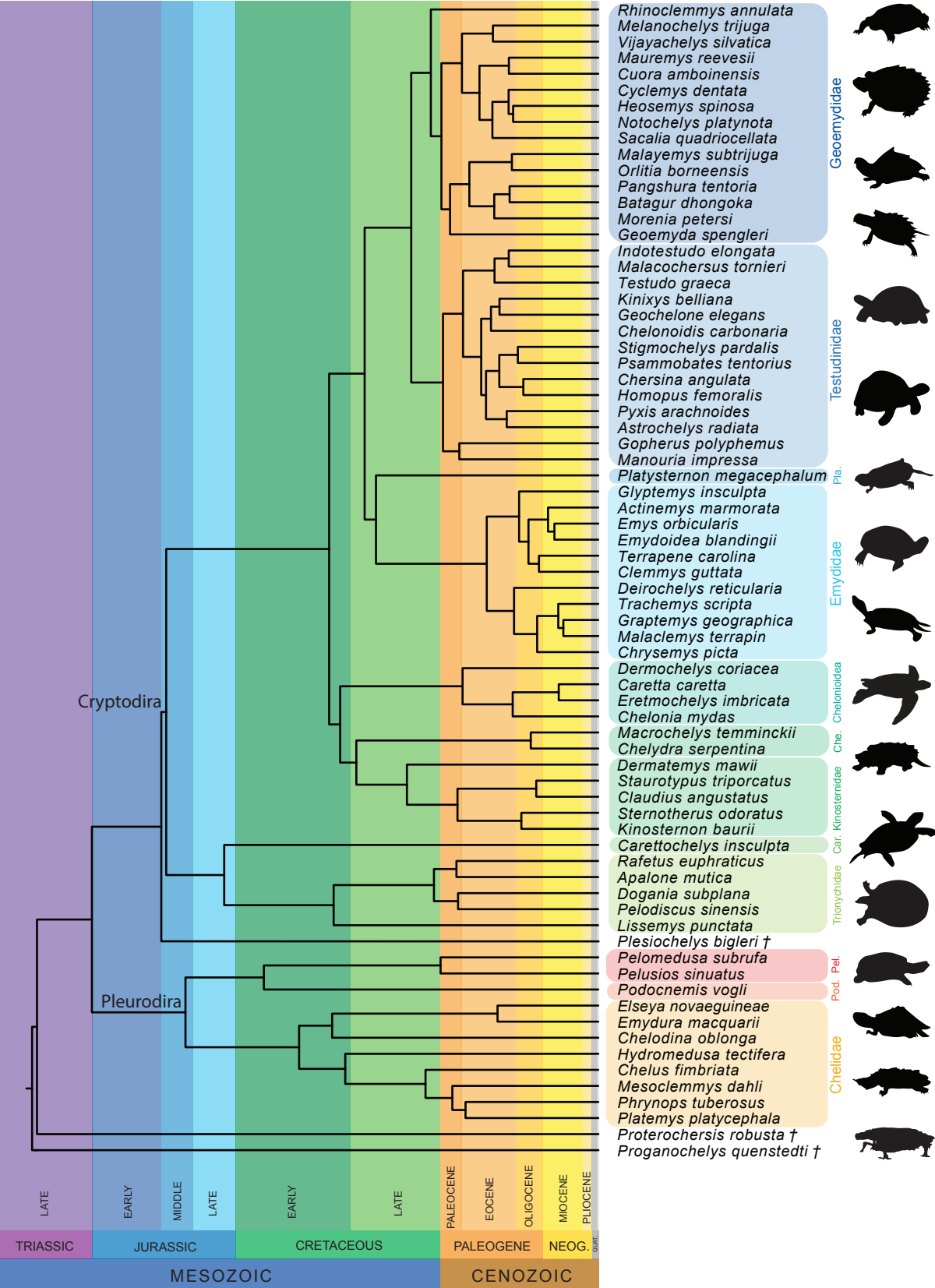

Supplement: Supplemental Information 5 — Abbreviation: Car, Carettochelyidae; Che, Chelydridae; Pel, Pelomedusidae; Pla, Platysternidae; Pod, Podocnemididae. [file peerj-08-10490-s005.pdf]

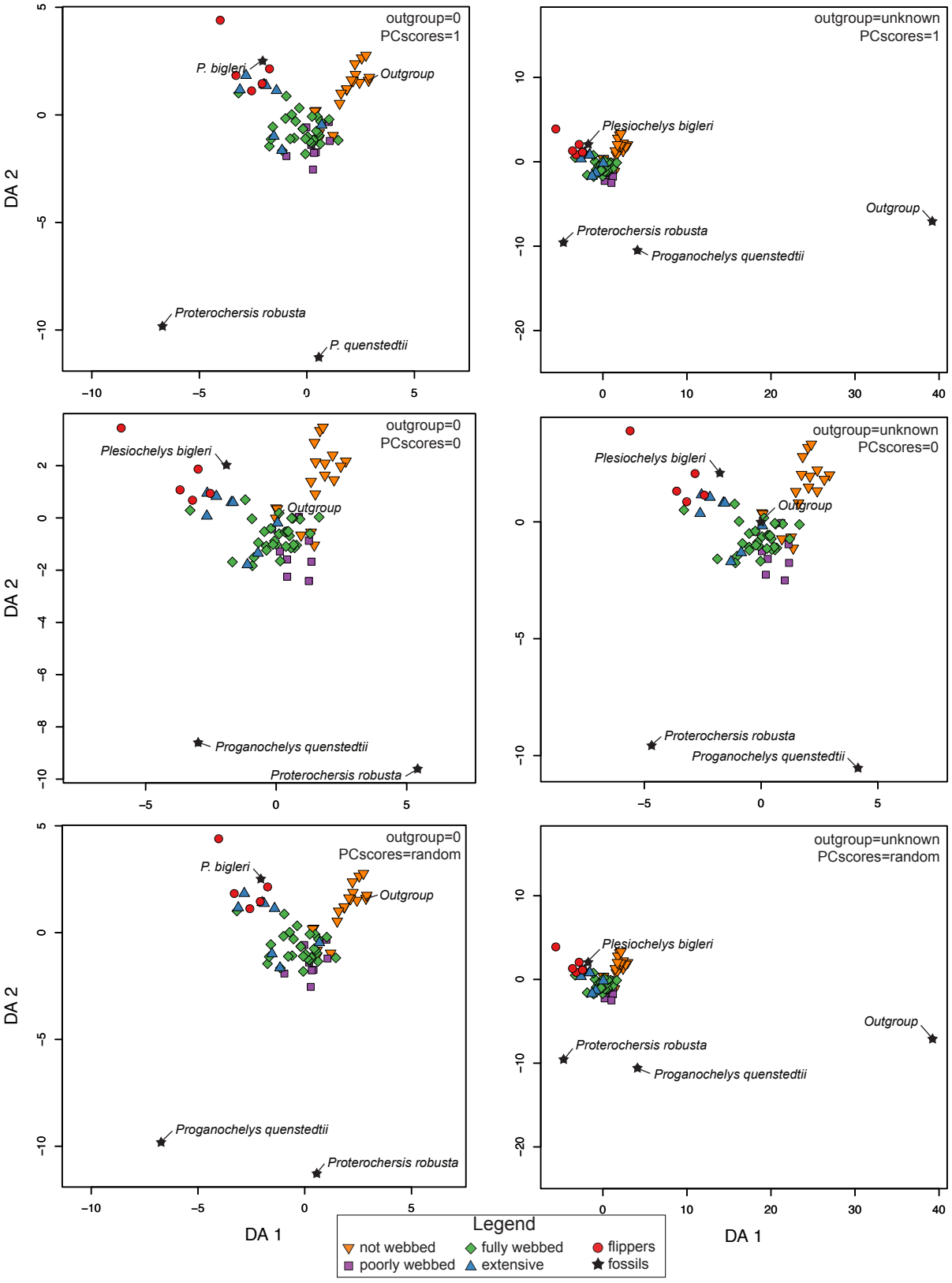

Supplement: Supplemental Information 6 — We tested ways to improve the results on fossil turtles by using an outgroup where all the PC Scores are equal to 0, 1, or randomized scores. The artificial outgroup was tested using two different categorizations, one identified as tnot webbed (category 1), and one as unknown, like the other fossils. Creating this outgroup did not improve the results of the analysis for the fossil turtles. Phylogenetic tree used in the analysis is available in Supplementary Files, Fig. S7. [file peerj-08-10490-s006.pdf]

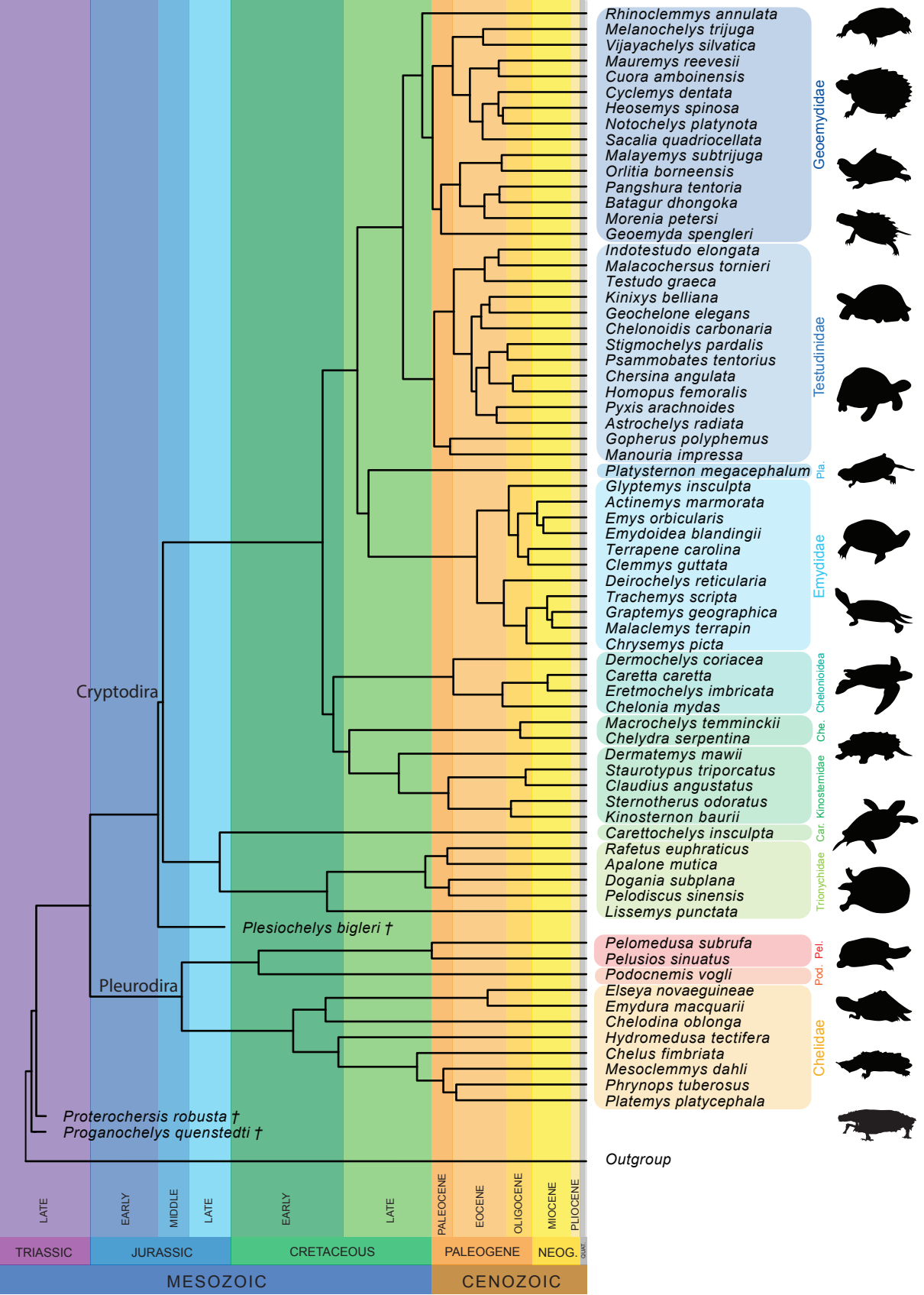

Supplement: Supplemental Information 7 — Abbreviation: Car, Carettochelyidae; Che, Chelydridae; Pel, Pelomedusidae; Pla, Platysternidae; Pod, Podocnemididae. [file peerj-08-10490-s007.pdf]
