# Supplementary material for: The ecomorphology of the shell of extant turtles and its applications for fossil turtles
Source: PeerJ. 2020 Dec 22;8:e10490. doi: 10.7717/peerj.10490 (PMC7761203; doi:10.7717/peerj.10490)
Supplement: Supplemental Information 8 — The number of genera for each major clade is based on the TTWG (2007). Abbreviations: Ntotal = number of known genera; Nsample = number of sampled genera. [file peerj-08-10490-s008.docx]

| **Clade** | **N_total_** | **N_sample_** | **percentage (%)** |
| --- | --- | --- | --- |
| **Carettochelyidae** | 1 | 1 | 100 |
| **Chelidae** | 14 | 8 | 57 |
| **Cheloniidae** | 5 | 3 | 60 |
| **Chelydridae** | 2 | 2 | 100 |
| **Dermatemydidae** | 1 | 1 | 100 |
| **Dermochelydidae** | 1 | 1 | 100 |
| **Emydidae** | 12 | 11 | 92 |
| **Geoemydidae** | 19 | 16 | 84 |
| **Kinosternidae** | 4 | 4 | 100 |
| **Pelomedusidae** | 2 | 2 | 100 |
| **Platysternidae** | 1 | 1 | 100 |
| **Podocnemididae** | 3 | 1 | 33 |
| **Testudinidae** | 17 | 15 | 88 |
| **Trionychidae** | 13 | 7 | 54 |
